# New York Letter

**Published:** 1892-03

**Authors:** 


### Correspondence.

### NEW YORK LETTER.

NEW YORK, February 17, 1862.

The dispensary question is again coming to the front here, and is exciting a great deal of comment in medical circles. The abuse of charity has attained such enormous proportions up to the present time, as to arouse grave concern in the minds of the larger number of medical practitioners of this city. Though there is but little outspoken condemnation among the doctors, there are significant complaints and mutterings on every side. It has been suggested to organize a "Practitioners Protective Association," the main object of which is to be, to induce those who manage dispensaries to restrict attendance of applicants, and to demand of them certificates from physicians or other qualified persons attesting proof of their *utter* inability to pay the ordinary physician's fee. Should this effort prove abortive, it is the aim of the society to commence an open and unremitting warfare against all dispensaries throughout the city. This would certainly be an extraordinary course to pursue, but it must be borne in mind that violent diseases require violent remedies, and there can be no question that free treatment, as carried out in this city, is one of the worst evils a young practitioner has to contend with. This question of free treatment will continue to be agitated here until struggling practitioners will not be deprived of the means of maintaining themselves and their families to the profit of the few who manage these dispensaries for their own interest and ambition.

A very interesting patient was presented to the Clinic of Dr. Janeway at Bellevue Hospital, a few days ago. He was a young man who had had a respiration of 152 a minute for the past three years. He was formerly on the police force of Boston, and in the fall of 1889, while riding on the platform of a car, was thrown violently on the ground, falling on the back of his head and shoulders. He became at once unconscious, and remained so for ten days afterwards. The case was considered by the physicians who saw it as one without precedent in medical records.

Dr. Van Valsah read a paper last night, before the Section on Practice of the New York Academy of Medicine, on "The Causation and Treatment of Chronic Diarrhœa," and summed up with the following conclusions:

1st. Remove the cause.

2nd. Improve the nutrition and conserve the energy of the patient.

3rd. Secure active elimination and prevent infection.

4th. Cleanse the alimentary canal and keep its contents sweet.

5th. Secure perfect digestion of the food.

6th. Promote absorption and diminish the work of the diseased parts.

Dr. A. H. Smith, in discussing the paper, said there were a few etiological factors occasionally met with in chronic diarrhœa to which the author of the paper had not referred, and one was the malarial element. Every now and then he met with in hospital practice, cases which baffled his efforts at finding a causation, and where the most carefully studied treatment failed to control the course of the diarrhœa, and yet was at once arrested by the exhibition of an antiperiodic. Just how the malarial poison produces the diarrhœa, he was unable to say, but it was a fact which every one could verify at the bedside.

Speaking in a general way of the treatment of diarrhœa, he thought animal food was very much better than farinaceous for people with this malady, and the reason was that rest is obtained more effectually by throwing the digestive duty on the stomach, inasmuch as animal food is principally digested and absorbed from that organ. Farinaceous food, on the other hand, has to pass the pilorus, and be brought into relation with the fluid of the duodenum before it is in condition for absorption. The indication for the use of animal food was when intestinal digestion was imperfect. Where they had a fatty diarrhœa, he considered ether to be a valuable remedy, as by its use the function of the pancreas was stimulated, and in that way it exerted a beneficial effect.

Dr. Quinby advised cleaning out the intestinal canal in cases of chronic diarrhœa. It was with great fear and trembling that he commenced treating by means of a cathartic patients who had ten or twelve movements of the bowels a day, but he had never since regretted doing so. He did not wish to be understood as saying that these cases should be treated persistently and permanently in this way, but intermittently. He has had great benefit from large doses of pancreatine in the treatment of the diarrhœa of tubercular patients. He has seen cases where this form of diarrhœa was checked up to the very hour of death, and the colicky pains entirely relieved by large doses of this drug administered every hour or hour and a half after eating.

The trial of a young medical student in this city on a charge of poisoning his wife with an overdose of morphine has aroused a great deal of attention in consequence of the interesting questions involved in the medical jurisprudence of the case. The medical experts on both sides flatly contradicted each other on essential points, making clear to the public the necessity for impartial scientific commissions, appointed by and responsible only to the court, as opposed to the present custom, where medical experts are avowedly employed to take one side or the other, and thus hopelessly muddle the minds of both judge and jury.

The evidence in this case clearly pointed to morphine poisoning, though one of the medical experts for the defense swore that the symptoms were those of hemorrhage of the pores instead of morphine narcosis. To judge from the testimony of the medical experts, taken on the trial, it would appear to be impossible for any one to arrive at a definite conclusion as to the cause of death in the case. The doctors, as is usually the case, differed from each other in almost every instance, as far as their mutual interests were concerned, and though from a medical standpoint they did not help the jury very much, a verdict of murder in the first degree was found against the prisoner.

Dr. J. A. Wyeth is the most enthusiastic advocate of cocaine in its application to minor surgical operations of any surgeon in New York. All surgical operations of a minor character at the New York Polyclinic are performed under the use of this drug to the complete exclusion of chloroform or ether, at least in the service of Dr. Wyeth. He has developed the technique of cocaine anæsthesia in so perfect a manner that he can cut into a mammary abscess, remove a conical stump of bone, or perform the operation of urethrotomy by the use of this drug without the slightest discomfort to the patient, and with the utmost satisfaction to himself.

